# Nonunion of a posterior glenoid rim fracture leading to posterior subluxation

**DOI:** 10.4103/0973-6042.59974

**Published:** 2009

**Authors:** James R. Barnes, Navraj Atwal, Partha Sarangi

**Affiliations:** Department of Orthopaedic, Bristol Royal Infirmary, Bristol

Sir,

Fractures of the glenoid cavity account for 0.1% of all fractures. Of these, only 1 in 10 are substantially displaced requiring surgical intervention [1‐5].

Fracture of the posterior glenoid rim is rare. Although this fracture has been reported, we are unaware of any previous report that this type of fracture has gone on to develop a non-union.

A 36-year-old man was referred to the shoulder clinic by his general practitioner with symptoms of persistent pain and loss of motion following an injury.

The initial mechanism of injury was a fall onto an outstretched hand, associated with immediate pain and deformity of the shoulder. The patient was able to correct the deformitywith gentle pressure from his contralateral hand. Antero posterior (AP) and lateral radiographs taken in the Emergency Department at the time did not identify any abnormality. A diagnosis of a possible shoulder dislocation was made, and arrangements were made for physical therapy. After initial improvement, the patient noted increasing pain and loss of movement.

In the shoulder clinic, it was noted that there was global wasting of muscles around the right shoulder and localized tenderness over the anterior shoulder capsule. All movements of the shoulder aggravated the pain. Range of motion was restricted to 20° external rotation and 100° forward elevation. AP and axillary views at this stage revealed no abnormality. An magnetic resonance imaging (MRI) scan revealed slight posterior subluxation of the shoulder with edema around the posterior glenoid edge, consistent with an undisplaced fracture of the glenoid.

A clinical diagnosis of posttraumatic adhesive capsulitis was made. Arthroscopic examination revealed extensive inflammation within the gleno-humeral joint and scar tissue within the rotator interval and anterior shoulder capsule. No anatomical abnormality was seen in the posterior aspect of the shoulder, so an anterior capsular release was undertaken.

Following surgery symptoms improved, particularly with regard to regaining movement.

His symptoms of activity-related pain persisted and it became apparent that there were signs of posterior shoulder instability. Radiographs of the shoulder, including axillary projections, were again inconclusive.

**Figure 1:**
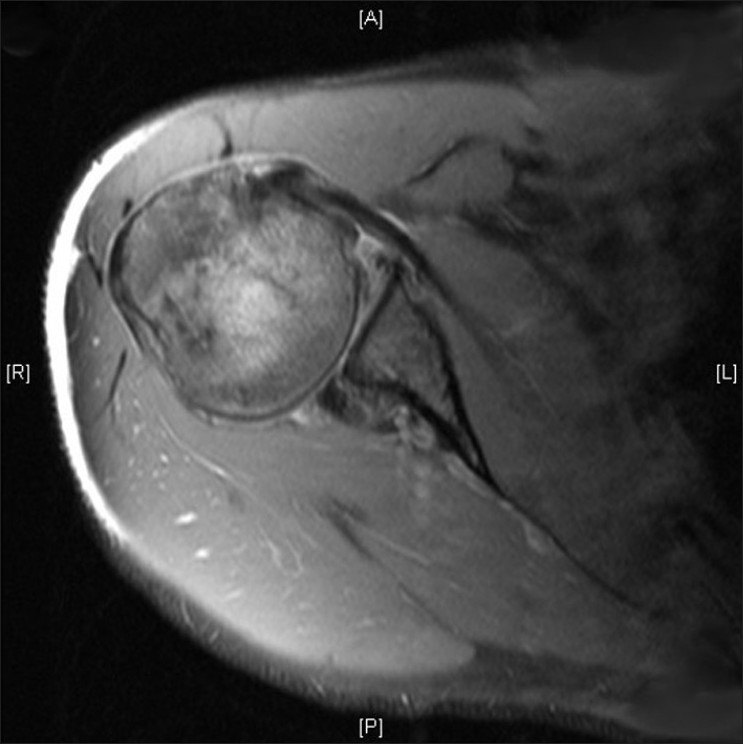
MRI showing glenoid fracture

A further MRI scan at this stage revealed a subtle posterior subluxation of the humeral head on the glenoid, with an associated articular rim fracture [Figure 1].

An open posterior stabilization of the shoulder was performed.

**Figure 2:**
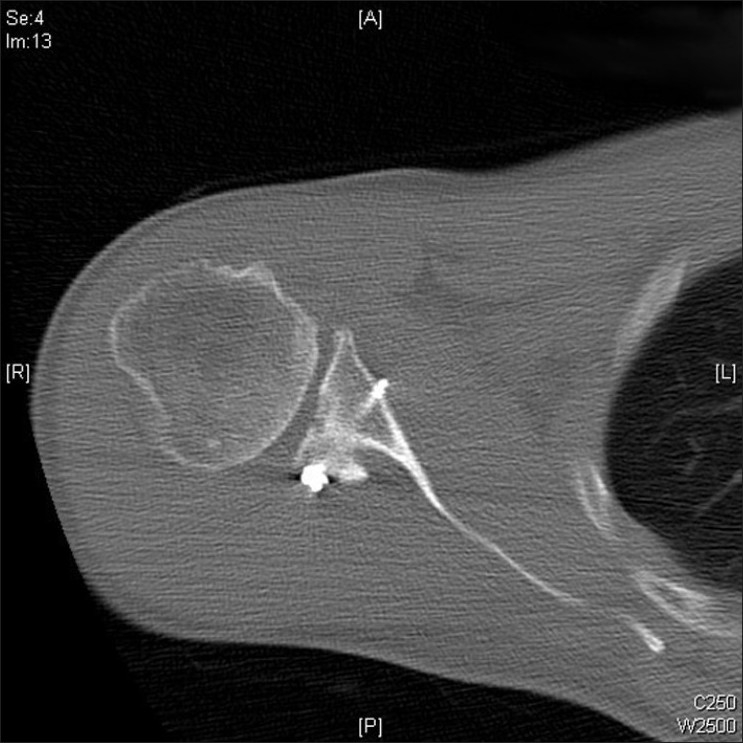
Computerised tomogrophy post-op showing position and fixation of iliac graft

Initial dissection revealed no abnormality of the posterior capsule or the posterior labrum. However, further dissection revealed that the posterior rim fracture was mobile, avascular and had failed to heal. It was removed, and a tricortical iliac crest graft was used to repair the defect. This was cut to shape and fixed flush to the posterior glenoid surface using 2 semi-threaded cancellous screws with washers. A stable reduction was achieved and the capsule and labrum were repaired [Figure 2].

The patient's shoulder was immobilized in an external rotation splint for 3 weeks before commencing physiotherapy.

**Figure 3:**
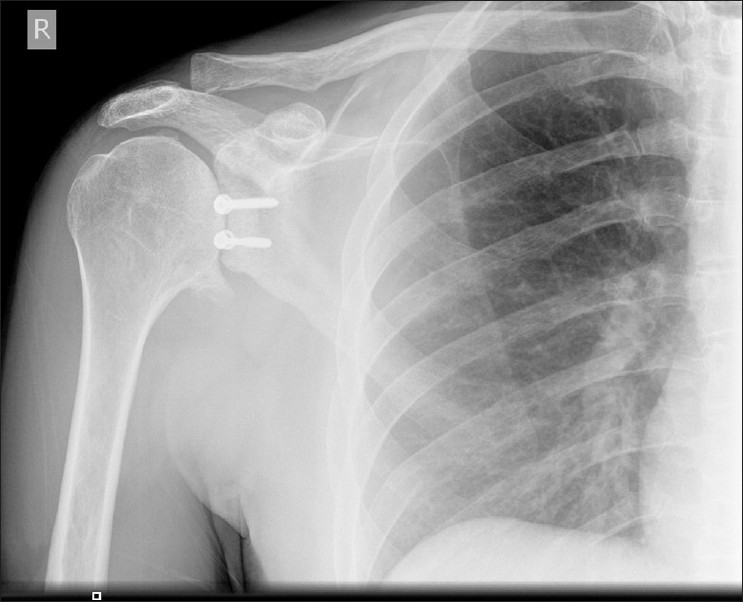
Antero posterior view showing progression of gleno-humeral joint arthritis

At 6 months following surgery, the patient's symptom of posterior instability had settled and he had regained movement (50° external rotation and 160° of forward elevation). Unfortunately the patient developed generalized pain, and repeat X-rays at this stage showed signs of early gleno-humeral osteoarthritis [Figure 3].

Given the extent of this patient's injury, we could really have expected him to have made a full recovery.

In this particular case, an undisplaced fracture of the posterior glenoid was not visible on  X-ray investigations. We believe that this fracture progressed to a non-union principally because the patient's shoulder was not immobilized and subjected to an early physical therapy program. The development of an anterior shoulder contracture pushing the humeral head posteriorly onto the fractured glenoid fragment may have contributed to the development of a non-union.

This case report describes the case of a gentleman who has already undergone 2 operations on his shoulder and will probably require a shoulder replacement due to evolving gleno-humeral arthritis. This may have been avoided by proper assessment and treatment at an earlier stage.

Dislocations of the shoulder are known to be associated with articular rim fractures.

Posterior dislocations of the shoulder should not be treated with early physiotherapy. They need to be immobilized.

When a suspected posterior dislocation of the shoulder presents to the Emergency Department the initial investigation should include an axillary view and subsequent follow-up in the fracture clinic.

If a patient continues to have persistent pain after a dislocation, then he/she requires early investigations with MRI or computerised tomogrophy.
